# Chronic Venous Insufficiency With Emphasis on the Geriatric Population

**DOI:** 10.7759/cureus.40687

**Published:** 2023-06-20

**Authors:** Harvey N Mayrovitz, Kawaiola C Aoki, Jessica Colon

**Affiliations:** 1 Medical Education and Simulation, Nova Southeastern University's Dr. Kiran C. Patel College of Allopathic Medicine, Davie, USA; 2 Medicine, Nova Southeastern University's Dr. Kiran C. Patel College of Allopathic Medicine, Fort Lauderdale, USA

**Keywords:** tissue dielectric constant (tdc), compression bandaging, cvi, compression treatment, lower extremity edema, peripheral edema, venous ulcers, geriatrics, venous disease, venous insufficiency

## Abstract

The underpinning of Chronic Venous Insufficiency (CVI) is valvular dysfunction, which manifests on a spectrum depending on the severity of insufficiency and duration of the disease. The mainstay of treatment relies on compression therapy of a proper type and intensity. In older adults, special consideration must be taken during the patient encounter to account for age-related factors. This review discusses the clinical presentation, diagnosis, and mimicking of CVI, focusing mainly on older adults. The epidemiology, risk factors, disease burden, and grave complications ­- such as thrombosis and ulceration, are reviewed. The physiological impacts of CVI are described, providing the background for treatment strategies, including non-invasive, medical, and surgical therapies. The findings show advanced age to be an important risk factor contributing to CVI and that other age-related factors add to the risk of severe complications. Clinical assessments combined with objective measurements that assess localized skin water using tissue dielectric constant values or whole limb assessments may aid in the differential diagnosis. Furthermore, understanding the mechanism of action of compression therapy, the mainstay of CVI treatment, and its physiological impacts, allows for its informed use in geriatric patients with increased risks of potential compression-related side effects.

## Introduction and background

Chronic venous insufficiency (CVI) is more common in Western Europe, the United States (US), and other industrialized nations than in developing countries [1,2]. More than 25 million adults in the US are estimated to have CVI [3]. The many burdens of chronic venous insufficiency (CVI) include skin hyperpigmentation, eczema, and lipodermatosclerosis, in addition to lower extremity edema, cramping, pain, and the development of varicose veins [4-7]. Further, CVI is a common precursor of venous leg ulcer (VLU) development [8] that is associated with pain [9-11] and may be difficult to manage, especially in the elderly [12-15]. The linkage between CVI and VLU is demonstrated by a study in which more than 30% of patients who had a VLU also had CVI caused by combined venous obstruction and superficial venous reflux [16]. In that study, the most common independent predictor of CVI was a history of deep venous thrombosis (DVT). Factors contributing to CVI, such as reflux in superficial and perforating veins, increase with advancing age [17], as does the link between CVI and VLU [18]. In fact, about one-third of patients develop CVI from a varicose vein, with a rate of occurrence that increases with aging [19]. This review discusses multiple issues associated with CVI in the geriatric population. These include its (1) incidence, (2) clinical presentation, (3) diagnosis and imaging, (4) lower extremity conditions that mimic aspects of CVI such as lymphedema and lipoedema, and (5) to address the specific risk factors and pathophysiology in the geriatric population with (6) detailed considerations of compression physiology and other treatment modalities for CVI.

## Review

This review is in part based on information derived from an analysis of published material obtained via literature searches of four major electronic databases and, in part, based on professional experiences and original material of the senior author (HNM). The databases searched were PubMed, Web of Science, EMBASE, and Biomedical Reference Collection: Comprehensive. The primary search term strategy for each of these was as follows. The title terms used were “venous insufficiency”, “venous disease,” and “CVI”. These phrases were individually searched when combined (AND condition) with the following terms if they appeared in the abstract; geriatric, elderly, old age, older, aging, or compression. Retrieved titles were first screened for potential relevance, followed by an abstract review for further clarifications if warranted by the title. Articles that were deemed relevant were retrieved and reviewed. In some cases, the bibliography of the retrieved articles provided additional sources. Supplemental searches were done as needed.

Epidemiology of CVI

CVI prevalence depends on the specifics of the population, geographical location, classification, and methodology used. Most studies report it more prevalent in women [2,3,19-21], but at least one study reported it more prevalent in men [21]. Further, sex differences appear to depend on the age group being evaluated, with CVI prevalence overall varying from <1% to 40% in women and <1% to 17% in men [21]. However, after age 55, it is more prevalent in men [22]. The source of the CVI in one-third of cases follows an initial diagnosis of varicose veins that increases with age [19]. There appears to be an impact of the geographic region with CVI prevalence that is greater in Western Europe, the United States, and other industrialized nations compared to undeveloped regions [1,2]. In the United States, there are more than 25 million adults with CVI, with about six million having advanced CVI [3]. International CVI prevalence assessed in 99,359 persons was reported as 29.9%, 26.6%, 24.9%, and 19.8%, respectively, in Eastern Europe, Latin America, Western Europe, and Asia [23].

Clinical presentation

**Figure 1:**
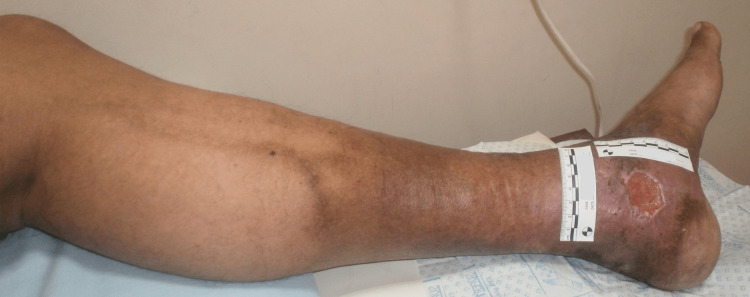
Skin changes associated with chronic venous insufficiency (CVI) Skin hyperpigmentation in the area of the involved veins is shown together with a venous ulcer that developed as a consequence of the patient’s CVI. Figure courtesy of Dr. HN Mayrovitz.

The signs and symptoms depend on the severity and duration of the pathology contributing to the disease. Chronic Venous Disease (CVD) includes a spectrum of conditions, from benign spider veins to cutaneous changes and ulceration. A common early sign of CVD is the presence of telangiectasias (spider veins) and reticular veins [21]. In the presence of valve dysfunction, these veins may widen due to blood reflux, causing them to become more tortuous and transform into varicose veins [24]. Clinical features of CVI may include dilated veins, edema, discomfort, pain, and skin changes associated with hemosiderin being deposited in the skin, along with skin hyperpigmentation and eczematous dermatitis. An example of skin changes is shown in Figure 1 of a patient that has also had a VLU near his medial malleolus as a consequence of his CVI.

**Figure 2:**
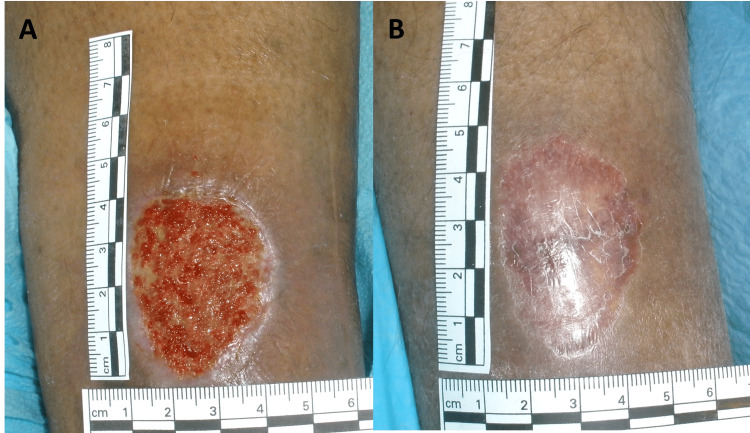
Venous ulcer attributable to Chronic Venous Insufficiency Part A) Initial ulcer after debridement showing granulation tissue, Part B) After 12 weeks of treatment including compression bandaging. The figure is courtesy of Dr. HN Mayrovitz.

Patient-reported symptoms include leg heaviness and aching or cramping pain in about half of patients [25-27]. Discomfort is generally made worse due to prolonged standing and generally made better with leg elevation. In about 20% of patients, other reported symptoms include pruritus or paresthesia [25]. Areas near the involved veins may become tender or may develop superficial thrombophlebitis-with painful, indurated, and inflamed areas along involved veins with the possibility of painful venous claudication due to obstruction of the deep venous system [28]. As the duration and extent of CVI increase, lipodermatosclerosis may occur due to subcutaneous fat inflammation [29]. This is associated with the skin, subcutaneous tissue, and deep fascia becoming indurated, adherent, and hardened [30]. Advanced CVI is a significant risk factor for developing lymphedema and cellulitis and is a major cause of venous ulcers [31]. A venous ulcer due to CVI is shown in Figure 2 as it looked initially and after healing from 12 weeks of treatment, including compression bandaging.

Risk factors for chronic venous insufficiency

Risk factors for CVI include advanced age, being female, having been pregnant, obesity, prolonged standing, and immobility, and in geriatric patients, advanced age is the most prevalent risk factor [1,3,32]. This age dependence is in part due to a correlation between advanced age and increased venous wall deterioration, and increased venous pressure due to weakened calf muscles with age [1]. One study reported that for each one-year age increase, the CVI risk increases by 6% [33]. The greater risk due to prior pregnancy is partly due to the elevated venous pressures that lead to venous valve injury, dysfunction, or failure and partly due to the action of the hormone relaxin on venous valves [2,34]. The manifestation of these changes progresses with increasing age. Similar impacts on venous valves, as precursors to CVI, occur in elderly persons who have a BMI >30 Kg/m2 [35], suggesting that geriatric adults with high BMIs are more likely to have a greater predisposition to CVI. CVI is also more prevalent in persons with occupations that require prolonged standing [36]. Multiple studies indicate that prolonged sitting or standing contributes to CVI [37-39], likely due to a sustained increase in venous pressure [38]. As a result, geriatric persons who had previously been employed in occupations requiring an extended amount of sitting or standing are likely at greater risk of CVI.

Diagnosis based on CEAP classification

The CEAP classification system derives from the words Clinical, Etiologic, Anatomic, and Pathophysiologic [40]. A summary of the CEAP classification is shown in Table 1. A CVI diagnosis requires a classification of ≥ C3 on the “C” scale [20]. Other than the clinical presentation (“C”), CEAP does not assess disease severity but can be used to estimate the venous severity scores, which can be used to monitor changes in the patient’s condition [41].

**Table 1:** CEAP Classification of Chronic Venous Disease (CVD) The table is a compilation of information from references 20, 24, 28, 40, and 42. In the table, the following notes apply. * The subscript “r” can denote recurrence, for example, “C2r” for recurrent varicose veins or “C6r” for recurrent, active venous ulcers. † Telangiectasias considered < 3 mm diameter; varicose veins considered > 3 mm diameter ‡ Clinical class C3 and above represents Chronic Venous Insufficiency

C		E		A		P	
Clinical*		Etiology		Anatomy		Pathology	
C_0_	No visible signs	E_c_	Congenital	A_s_	Superficial	P_r_	Reflux
C_1_	Spider veins, telangiectasias, or reticular veins^†^						
C_2_	Varicose veins without clinical signs of CVD^†^	E_p_	Primary	A_d_	Deep	P_o_	Obstruction, thrombosis
C_3_	Varicose veins with edema^‡^						
C_4_	Varicose veins with skin changes^‡^ C4a: Pigmentation, purpura, eczema C4b: Lipodermatosclerosis, atrophie blanche C4c: Corona phlebectatica	E_s_	Secondary E_si_: Intrinsic E_se_: Extrinsic	A_p_	Perforator	P_r,o_	Reflux and obstruction
C_5_	Healed venous ulcer^‡^	E_n_	Not identified	A_n_	Not identified	P_n_	Not identified
C_6_	Active venous ulcer^‡^						

Noninvasive diagnostic methods

Non-invasive testing may be used to categorize the etiology, anatomy, and pathophysiology (“EAP”) factors further to characterize the extent of the condition [40,42]. Duplex ultrasound is currently the gold standard, but other diagnostic tests may be helpful in particular circumstances [43].

Doppler Ultrasound

Duplex ultrasound assesses venous system structure and function in suspected CVI. It combines B-mode imaging to visualize deep and superficial veins with pulsed Doppler blood flow assessments to detect, localize, and evaluate valvular incompetence and venous obstruction [42]. Venous blood flow absence or disturbance due to deep vein thrombosis or venous stenosis may be directly observed or inferred from blood flow features [28]. Standardized evaluations include assessments of the posterior tibial, popliteal, superficial femoral, common femoral, and greater saphenous vein for patency and valvular competence [44]. Valvular competence and reflux can be evaluated using a Valsalva maneuver [28] or a rapid cuff inflation-deflation technique [45,46]. Venous blood flow reflux can be detected by an inversion of the blood flow color pattern - blue flow toward the heart or red toward the periphery. Any significant flow toward the feet suggests reflux [28,42]. Venous incompetency extent is assessed by the duration of reflux, with longer reflux times suggesting more severe disease but not necessarily worse clinical outcomes [47,48]. A limitation of duplex ultrasound is that it cannot assess pelvic veins [20].

Computed Tomography (CT) and Magnetic Resonance Imaging (MRI)

CT and MRI are standardized, reproducible, and non-invasive methods but may be less accessible than ultrasound [49]. However, both methods are superior in evaluating pelvic veins for the presence of venous obstruction and can also detect changes in the skin, subcutaneous fat, muscles, tendons, periosteum, and bones [20,49,50]. The use of intravenous (IV) contrast material may be indicated to visualize better deeper venous structures, perforating veins, and other venous malformations [28]. However, contrast is contraindicated in some older adults if kidney disease is present [51]; however, if used, the contrast administration can be timed through multiple scans so that different veins opacify depending on their blood flow and allow for sequential views [42]. In advanced CVI, which is more likely present in older persons, osseous changes and soft tissue calcifications are better visualized by CT, with lower extremity dermal fibrosis seen as hyperdense condensation [49].

In comparison to CT, MRI provides multiplanar images with a high spatial resolution for soft tissue and high sensitivity to detect subcutaneous edema and other changes. On T1-weighted images, periosteal hyperostosis is hyperintense, while subcutaneous fat is hypointense due to fibrosis. Metaplastic calcifications in cutaneous fat can also be visualized. On T2-weighted images, edema appears hyperintense due to water content [49]. Magnetic resonance venography (MRV) can visualize the vascular system without needing nephrotoxic contrast media [52]. Compared to CT or duplex ultrasound, MRV is reported to be superior in determining overall thrombus burden in smaller branching veins and diagnosing pelvic vein thrombosis [42].

Conditions that may mimic aspects of CVI

Conditions that may mimic aspects of CVI include cellulitis, lymphedema, thyroid dermatopathy, volume overload edema, and lipoedema.

Cellulitis

Cellulitis is a bacterial infection of dermal and subcutaneous tissue that disrupts the skin barrier [53,54]. Patients with cellulitis often have inflammatory signs that mimic those seen in CVI, including skin warmth, erythema, edema, and pain. In older populations, pseudo cellulitis, a condition mimicking cellulitis or stasis dermatitis, may occur [53,55]. Pseudocellulitis in older persons must be diagnosed promptly to avoid complications and the higher risk for antibiotic-related adverse events [56]. As cellulitis is usually unilateral, a bilateral presentation is more suggestive of venous insufficiency or stasis dermatitis [57,58]. CVI and lymphedema are two major risk factors for causing lower-extremity cellulitis, with CVI nearly doubling the odds of cellulitis [58]. When venous and lymphatic drainage is obstructed, the clearing of microbes is prevented, facilitating local skin infection and potentially spreading through superficial tissue [57].

Lymphedema

**Figure 3:**
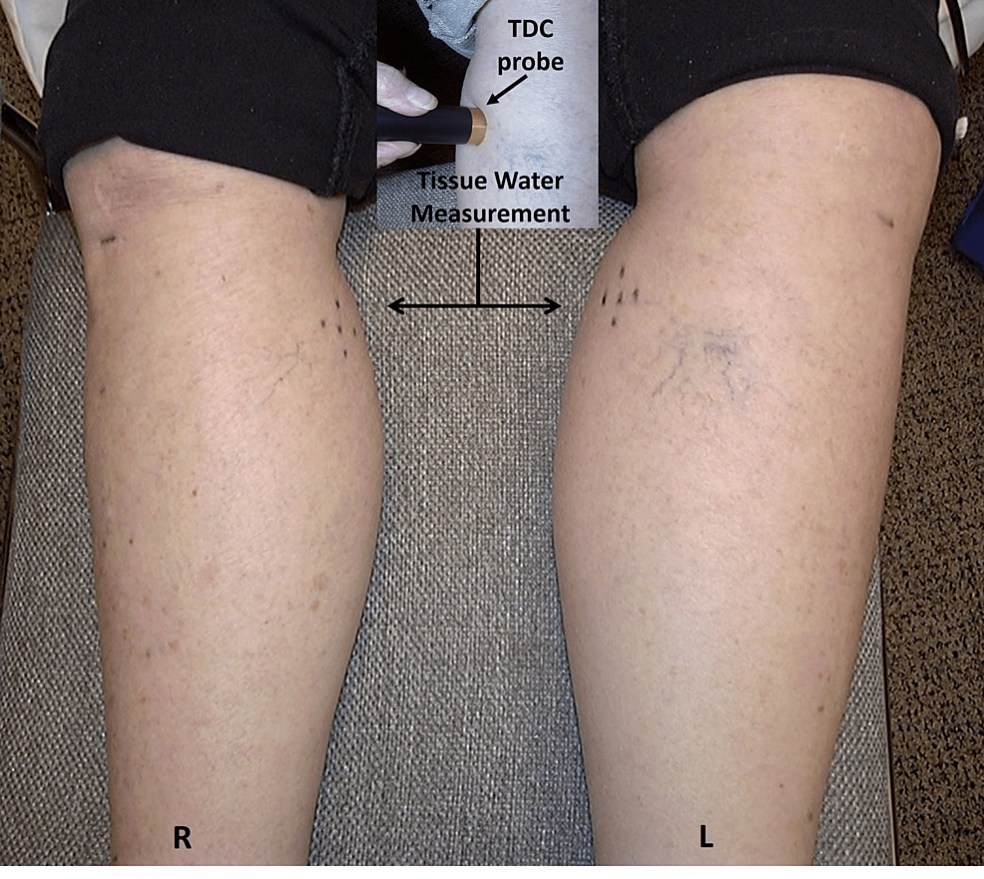
Measuring tissue water via tissue dielectric constant (TDC) values TDC, as an index of skin-to-fat tissue water, is shown in the inset being measured on the left leg of a 77-year-old female with unilateral lymphedema 5-years after surgery for gynecological cancer. The TDC value on the lymphedematous left leg at the site with the five dots was recorded as 48.7 in comparison with a value of 24.2 on the non-affected right leg. The figure is courtesy of Dr. HN Mayrovitz.

Impairment of lymphatic drainage of the lower extremities causes protein-rich fluid to accumulate in tissue spaces that result in unilateral or bilateral edema, pain, atrophic skin changes, and in some cases, secondary infections [59]. In advanced stages, the overlying skin may appear orange-peel-like (peau d’orange) or thickened and leathery [60], and in very advanced stages, the skin becomes hardened and appears with cobblestone-like papules, plaques, and nodules [61]. Primary lymphedema is due to developmental abnormalities of lymphatic vessels, valves, or nodes and may develop congenitally, around puberty, or as an adult, and is impacted by age-related lymphatic deterioration [62]. Secondary lower extremity lymphedema is often due to cancer-related surgery or radiation treatments that diminish lymphatic drainage function [63,64]. Lymphedema is diagnosed by clinical assessments, although quantitative measures based on bioimpedance spectroscopy [65] and localized measures of tissue water using tissue dielectric constant (TDC) values [66-69] are now available. TDC values can be used to assess localized edema at almost any site due to any cause. A probe is shown in Figure 3 measuring TDC in a 77-year-old woman with unilateral lymphedema caused by gynecologic cancer treatment. Her affected left leg volume was 1370 ml greater than the right.

**Figure 4:**
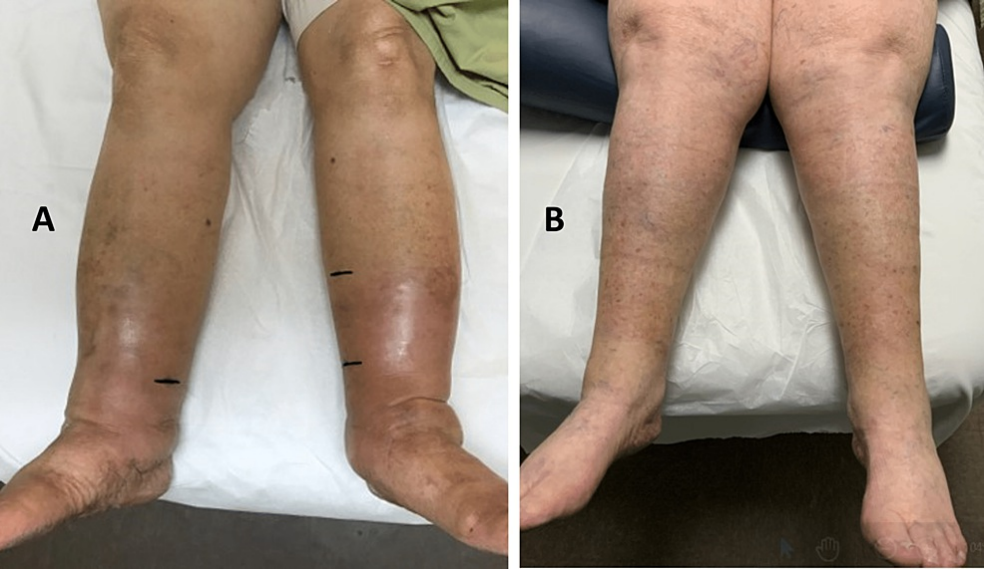
Bilateral chronic venous insufficiency (CVI) and Lymphedema In A is shown a 71-year-old female with CVI that has impacted both legs. Measurements of TDC at 8-cm proximal to the medial malleolus show a TDC value (as an index of tissue water) of 67.3 compared to a non-edematous forearm site of 25.0. In B, is shown a 71-year-old female with long standing bilateral lymphedema. Measurements of TDC at 8-cm proximal to the medial malleolus show a TDC value of 51.2 compared to a non-edematous forearm site of 25.2. The figure is courtesy of Dr. HN Mayrovitz.

Other potentially age-related issues of older adults related to developing lower extremity lymphedema include total hip or knee replacement procedures and prostate surgery. If CVI is present before surgery, a fully functional lymphatic system helps compensate for co-present CVI effects. But, if after surgery or radiation treatment, the lower extremity lymphatics can’t handle the associated elevated interstitial fluid load, an acceleration in lymphedema is likely due to the prior CVI [70]. Consequently, differentiating between lower extremity lymphedema and CVI is sometimes problematic but not always. Figure 4 illustrates two bilateral cases, one in an 89-year-old woman with CVI and one in a 71-year-old woman with bilateral lymphedema.

Thyroid Dermatopathy

Another condition potentially mimicking some attributes of CVI is thyroid dermatopathy (TD), also known as pretibial myxedema, which is caused by thyroid dysfunction and is often seen in advanced age [71]. Hypothyroidism, neoplastic disease, and autoimmune disorders are more common in the geriatric population [72]. In older adults, Hashimoto’s thyroiditis is one cause of primary hypothyroidism [73]. TD usually presents with non-pitting edema and induration due to deposits and accumulation of glycosaminoglycans in the papillary dermis that subsequently extends to the reticular dermis [74,75]. The visual and feature similarity between TD-related lower extremities has been well illustrated [76]. Differentiating TD-related lower extremity edema from CVI is aided by clinical history and histology [77], but since thyroid dysfunction signs and symptoms may mimic those of aging, a TD differential may be missed [71,78].

Lower Extremity Edema from Volume Overload

**Figure 5:**
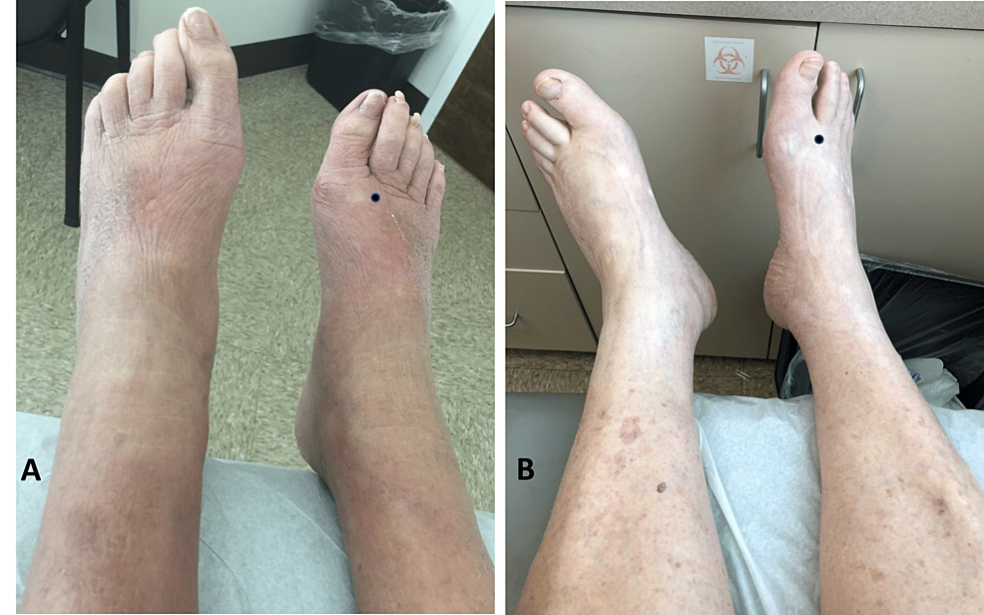
Lower extremity edema in congestive heart failure Part A shows a 75-year-old male who has class II heart failure with preserved ejection fraction (HFpEF). Part B shows an 89-year-old man who also has class II heart failure but with reduced ejection fraction (HFrEF). The black dots on the foot dorsum are sites at which TDC was measured to assess tissue edema. For patient A, a TDC value of 47.0 was measured vs. a value of 24.3 in non-edematous tissue. For patient B, a TDC value of 40.8 was measured vs. a value of 24.7. The figure is courtesy of Dr. HN Mayrovitz. in non-edematous tissue.

Many chronic diseases are associated with lower extremity edema; these include congestive heart failure (CHF), renal insufficiency, cirrhosis, and pulmonary hypertension [79]. These most often cause lower extremity edema that is bilateral, pitting, non-tender, and usually without skin changes [80]. An example of two geriatric patients with CHF is shown in Figure 5. Both patients had their edema extent assessed on the foot dorsum using TDC measurements that showed significant excess tissue water in both cases. Patients with CVI also have dependent edema that improves with elevation but often have brawny, reddish skin changes due to hemosiderin deposition, as illustrated in Figure 4A [2,81]. In comparison, edema due to low plasma oncotic pressure in liver failure, nephrotic syndrome, and protein malabsorption does not improve with positional changes. As in thyroid dermatopathy, the symptoms of the above disorders may be confused with symptoms of aging and add to diagnosis uncertainty.

Lipoedema Mimicking CVI-related Swelling

Lipoedema is a chronic progressive disorder of adipose tissue accumulation, which may be mistaken for other pathologies that involve lower extremity enlargement. TDC measurements [82] or high-resolution ultrasound may differentiate this condition from lymphedema [83]. The pathophysiology of lipoedema is not fully understood, but co-present lymphedema doesn’t appear to play a major role [84]. Lipoedema occurs almost exclusively in women and is associated with hormonal changes, often arising within a few years after puberty but rarely during pregnancy or menopause [85,86]. The onset is usually insidious, and the disease progresses gradually [87,88].

Consequently, older patients are more likely to have a long-standing disease, increasing the chances of comorbidities related to age, severity, and disease duration. Severe impairments from pain may further reduce physical mobility in this population [89]. Advanced stages of the disease may be associated with secondary lymphatic insufficiency, referred to as lipo lymphedema, or deterioration of the venous system, referred to as venolipoedema [86,90]. These complications add difficulty in making a proper diagnosis.

Psychosocial effects of CVI 

The quality of life for patients with CVI is impacted by their symptoms, treatment options, and frequent medical appointments. Beyond the fact that CVI can cause lower extremity pain, swelling, skin changes, and lead to infection and ulcer development, there is superimposed anxiety, depression, low self-esteem, and social deprivation [91,92]. The progression to venous leg ulcers, as discussed subsequently, amplifies psychosocial effects in relation to ulcer odors and excessive exudate that triggers further feelings of low self-esteem with social isolation and depression [93], and in older patients, further worsens quality of life [24]. Geriatric patients with CVI or its complications often depend on caregivers to help care for their symptoms, creating emotional and social burdens for both patients and caregivers [94]. CVI symptoms and its complications also affect work and leisure activities, with 42% of patients who developed venous leg ulcers indicating interference with work and leisure activities [95]. Thus, psychosocial effects and patient quality of life are important management considerations.

Predisposition to deep vein thrombosis and thromboembolism

CVI is strongly associated with venous hemodynamics, predisposing to thrombotic events such as superficial or deep venous thrombosis (DVT) [96]. Venous thrombosis is more common in geriatrics, especially those older than 70, with a greater DVT risk for increasing CVI severity [97]. DVT is a major risk of pulmonary embolism, the third most common cardiovascular cause of death, and in the elderly, is an immediate threat to their life [98].

CVI and its linkage to venous leg ulcers (VLU)

A study of 600 healthcare workers with a median age of 42 years reported that clinical CVI and venous reflux were present in at least one leg in 69.1% of subjects [99]. In a geriatric age range of 65-74 years, venous disease with or without VLU was diagnosed in about 5% of patients admitted to the hospital [100]. Further, of 141 patients over the age of 75 admitted to the hospital for chronic peripheral edema without dyspnea, 69% had CVI [101]. In patients with CVI, VLU is a complication in more than 18% of patients older than 65 [102]. Treatment of VLU in the elderly is complex [103], with significant socioeconomic and life functioning impacts [104]. It is evident that preventing the transition of CVI to VLU is a fundamental treatment goal [105]. Although the pathophysiology of the CVI-venous ulcer connection is well-studied, it is incomplete [106]. Altered aspects of the microcirculation are likely involved in this transitional process [107-109].

Physiological considerations of CVI and its pathological impacts

**Figure 6:**
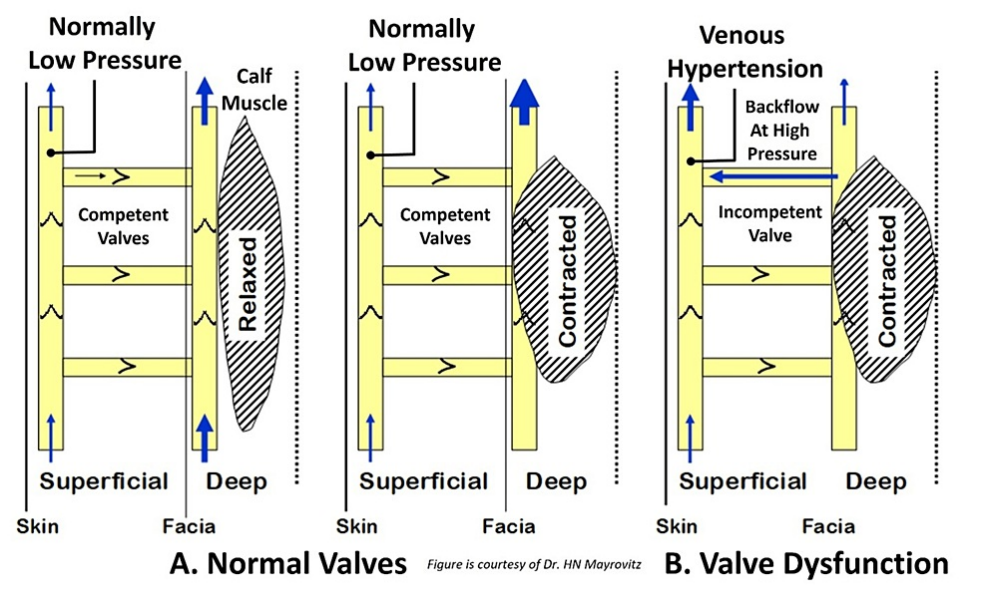
Impact and hemodynamics of incompetent venous valves In part A, the normally low pressure experienced by superficial veins becomes high pressure when valve incompetency is present as shown in part B. The high pressure causes venous injury that triggers an event sequence that may cause a venous ulcer.  Figure is provided as a courtesy of Dr. HN Mayrovitz

With legs in a gravity-dependent and relaxed position, lower extremity valves segment the hydrostatic pressure column, thereby reducing gravity-dependent pressure. Calf contraction acts as a pump by compressing veins; flow is directed centrally via valve presence. Walking vs. standing reduces dependent vein pressure by displacing volume centrally. Valve incompetence, as depicted in Figure 6, causes altered venous flow patterns that expose superficial veins to high impulse pressures and increased average venous ambulatory pressure. This venous hypertension is a major factor that leads to skin breakdown and ulceration. Normally, leg venous hemodynamics and volumes rely on valve competency of superficial, perforating, and deep venous systems to protect against gravitational and muscle pump pressures. Normal venous return for muscle is via the deep system and via the superficial system for skin and subcutaneous structures. Properly functioning valves in perforating veins prevent exposure of superficial veins to relatively high pressures in deep veins when they compress against fascia during calf muscle contraction. The properly functioning valves also permit unidirectional flow from superficial-to-deep veins during relaxation, resulting in an adequate blood volume ejection fraction to keep venous and leg volumes at normal levels. Valve dysfunction alters this situation. If perforator vein valves are dysfunctional, some deep vein volume at high pressure is transmitted to the superficial system with each calf contraction. As a consequence, effective ejection fraction for venous return from the deep system is reduced, excessive pressures in the superficial system further compromise valve competency, and the sustained increase in venous volume affects microcirculation causing endothelial cell changes and increased outward flux of fluids and materials from capillaries and venules. Changes in capillary hemodynamics, nutritional blood flow, interstitium content, and volume follow.

Compressive therapy as a therapeutic intervention

A mainstay of non-invasive treatment of CVI is compression therapy in the form of garments and bandages and, in some cases, intermittent pneumatic compression (IPC). Compression is used to reduce edema and thereby treat CVI or help heal VLU [110]. Of relevance to geriatric patients was a study of 102 patients with VLU who were divided by age above and below 65 years [111]. Of 51 geriatric patients, 25 were treated with moderate compression pressures from 37.1 to 46.3 mmHg, and 26 were treated with higher pressures from 61.1 to 72.4 mmHg. Younger and older groups healed better with higher compression pressures but complications such as skin superficial necrosis and discoloration were greater in geriatrics. Thus, it is important to consider aspects of compression therapy, including its functional dependence on compression materials, physiological rationale, mechanism of action, and impacts on limb pressures and blood flow.

Furthermore, if compression-induced tissue pressures become too large for too long, there is a negative impact on blood vessels and perfusion and on lymphatic vessels and their lymph flow [112]. Optimal tissue pressures have not been defined, and there is little direct information as to relationships between surface or sub-bandage pressures and associated tissue pressures [113]. Subdermal pressure measurements under compression garments give some idea of surface-to-subsurface radial pressure gradients to be expected at different sites [114]. Absolute pressures are greater at bony prominences, but gradients are larger for soft tissue. For example, measurements at the posterior mid-calf found that a sub-bandage pressure of 66 mmHg resulted in a subdermal pressure of 24 mmHg, whereas at the medial mid-calf, a sub-bandage pressure of 36 mmHg resulted in a subdermal pressure of 21 mmHg.

Compression in Relation to Function

**Figure 7:**
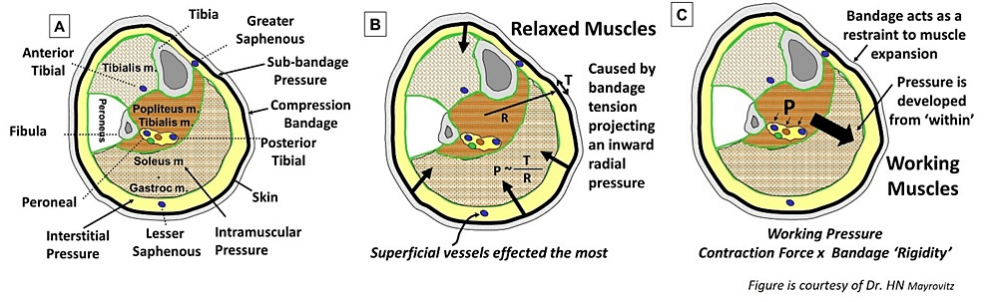
Illustrating main pressure effects of leg compression bandaging In part A, a leg cross-section with an effective radius (R) is shown with indicators of the various pressures. In part B, an inward-directed pressure (P) is caused by the tension (T) produced by the bandage. When the muscle contracts as indicated in part C as a working muscle, greater pressure is developed within the tissue called the working pressure, whose value depends on the contraction force’s magnitude and the bandage material’s relative rigidity.

Differences in garment or bandage materials produce functional differences [115-117]. For example, a material with a high percentage of elastic fibers forming the compression bandage is called a “long-stretch” bandage [118]. Stretch it more, and the recoil force increases. Such bandages stretch up to three times their zero-tension length on the leg and produce a sub-bandage pressure (SBP) that increases tissue, interstitial, and muscle pressures. It also alters the transmural pressure of blood vessels subjected to compression pressure, as shown in Figure 7. This SBP is directed radially inward and may be further distinguished as “resting pressure” or “working pressure” that distinguishes between a muscularly relaxed limb, illustrated in Figure 7B, from one undergoing muscular contraction, as illustrated in Figure 7C.

**Figure 8:**
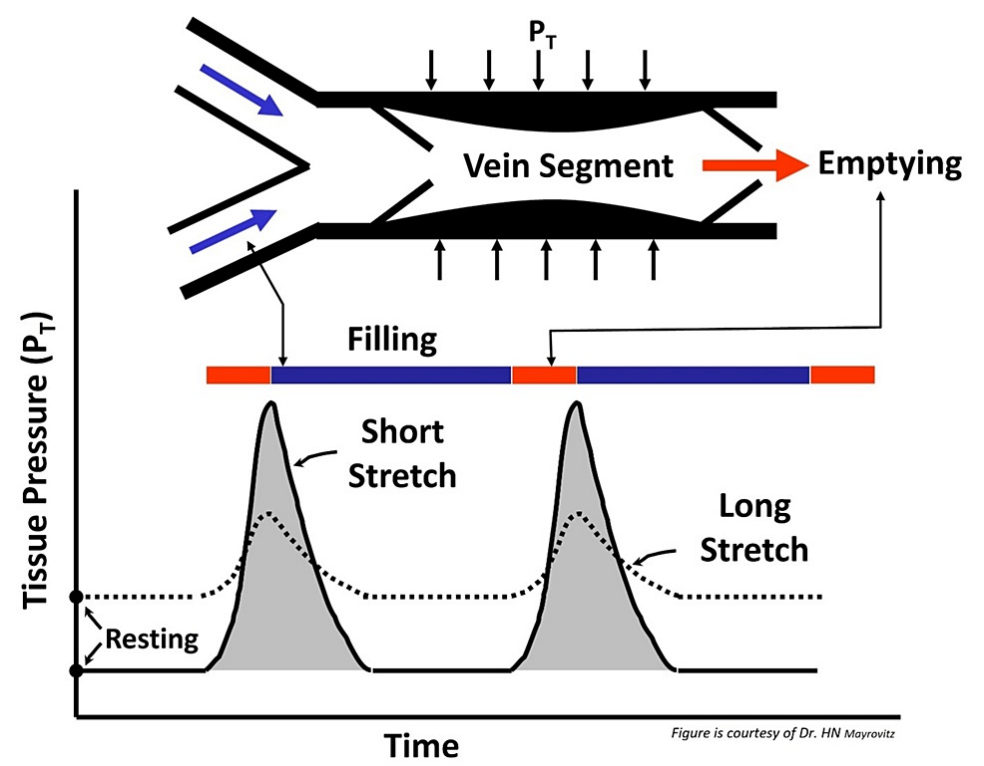
Tissue resting and working pressures with short and long stretch bandaging. Short stretch materials are stiffer, so, during calf muscle contraction, there is greater working pressure developed. During ambulation, this is more effective in moving venous blood centrally, thereby reducing venous pressure. The lower resting pressure of a short stretch bandage is because there is no need to tightly wrap it to achieve its function.

In contrast to the “long stretch”, a “short stretch” bandage has few elastic fibers and, under resting conditions, produces less recoil force on the leg, resulting in a lower resting pressure but a greater working pressure, as shown in Figure 8. Under resting conditions, a portion of the bandage-related SBP is transmitted interiorly and increases interstitial-tissue pressures (PT), which reduces vascular transmural pressures and helps reduce transcapillary filtration into tissue. Under dynamic working conditions, internal pressures assume much greater values when “no stretch” bandages are used since the effective dynamic compliance of the limb to volume expansion is reduced by the relatively more rigid bandage. These greater dynamic pressures are important in controlling edema via their favorable effects on interstitial fluid movement, which, together with lymphatic activation, helps reduce edema [119].

CVI and compression in relation to blood flow

**Figure 9:**
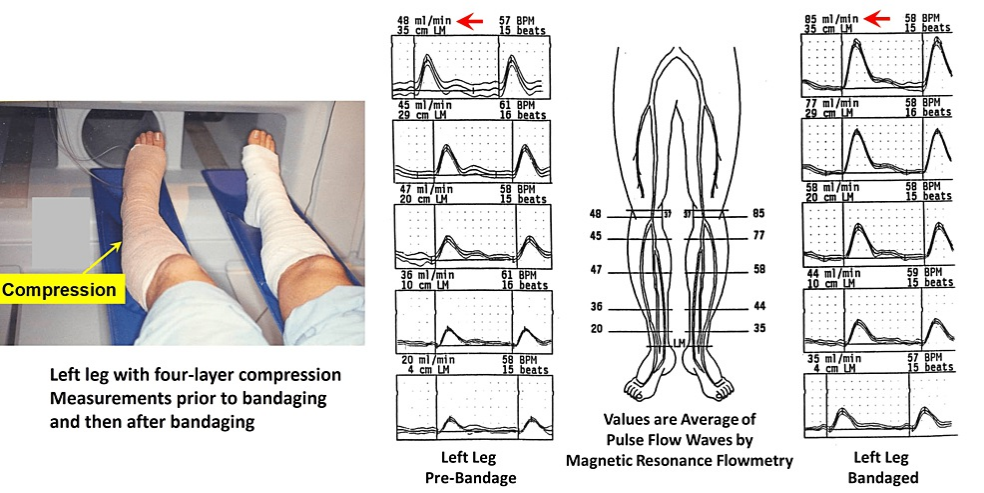
Impact of compression on leg pulsatile blood flow. The left panel shows legs with compression on the left leg about to enter into the magnetic chamber to measure pulsatile blood flow. The right panel shows pulsatile blood flow of the left leg prior to bandaging and then after bandaging. The vertical flow waveforms (ml/min) are obtained at varying distances from the lateral malleolus (LM). The main point is that the pulsatile flow is greater in the presence of compression at every location. The figure is courtesy of Dr. HN Mayrovitz.

Although the precise sequence whereby hemodynamic changes lead to skin ulceration is not fully worked out, evidence implicates reduced nutritional capillary density and degradation of capillary function [120,121]. These changes may be due to retrograde dynamic pressures transmitted to nutritive capillaries [122], causing trauma and inflammatory-like responses [123]. Surprisingly, in spite of increased leg blood flow in the ulcer region [124] and in peri-ulcer subcutaneous microcirculation, transcutaneous oxygen is reduced [108]. In addition to microcirculatory effects, limb compression augments arterial flow pulsatility [125], likely stimulating interstitial fluid and lymphatic dynamics and ulcer healing. Thus, appropriate compression therapy may preempt ulcer formation in cases of CVI and significantly aid in healing ulcers in part due to combined hemodynamic effects. The potential impact of compression bandaging on arterial pulses is visualized in figure 9, which shows the effects on leg pulsatile blood flow measured with a nuclear magnetic resonance method [125]. Pulsatile blood flow is seen to be substantially elevated at each longitudinal section where it was measured. The measurement locations are designated as cm proximal to the lateral malleolus (LM).

Other Treatments and Prevention Strategies Applicable to the Geriatric Population

Other treatments include exercise, the use of anticoagulants, surgical interventions, and ablation methods.

Exercise

In Europe, physical therapy is used for CVI but has not been widely adopted in North America [126]. As CVI progresses, ankle range of motion and calf muscle pump function is diminished. To investigate this, 31 geriatric patients with CVI were recruited and divided into control and treatment groups. Both groups received compression therapy, while the treatment group also received physical therapy focused on strengthening calf musculature. After six months, the treatment group’s calf muscle pump function increased with the structured exercise. Other studies indicate that improving calf muscle strength using tip-toe exercises or flexing and stretching the feet in the sitting position stimulates the calf muscle and improves venous blood pressure, residual volume fraction, and ejection fraction [127-129].

Anticoagulants

CVI is common among geriatric patients due to structural changes that occur in venous valves and walls with age, and CVI has been associated with a three-fold elevated risk of venous thrombosis and pulmonary embolism [130]. Treating venous thromboembolism in geriatric patients is complex since they have a higher likelihood of thrombosis and bleeding. Thus, if there are no contraindications, anticoagulant therapy may be prescribed [130,131].

Surgical Therapy

Surgical or endovenous procedures are used to treat CVI by removing an incompetent vein or isolating a reflux source from the rest of the circulation, an example of which is the classic saphenofemoral ligation and stripping procedure [20]. The great saphenous is ligated and dissected from the femoral veins along with its tributaries during this surgery. The lesser saphenous vein is dissected from the popliteal vein and ligated close to the junction. The role of superficial venous surgery was evaluated in advanced CVI in 146 legs treated with compression alone and 115 with compression and surgery [132]. After 12 months, the compression-only group had more legs with incompetent perforators than baseline, whereas the surgery group had significantly fewer legs with incompetent calf perforators. A study of 28 geriatric patients with CVI who were treated with superficial venous surgery concluded that surgery of the superficial venous system for treating CVI is effective and can be utilized for older patients with minimal risk [133].

Radiofrequency Ablation (RFA)

RFA uses electromagnetic energy to heat the vein wall, destroying the intima and resulting in a fibrotic occlusion of the vein [134]. This is done under local anesthesia in an outpatient setting. RFA was compared to the standard ligation/stripping procedure and reported earlier recovery and less postoperative pain [134]. RFA used to treat CVI due to superficial disease was evaluated in older patients and reported as safe and effective for geriatric patients with CVI [135].

Endovenous Laser Ablation (EVLA)

ELVA delivers laser energy directly into the lumen of the vein, causing the blood inside to boil and form steam bubbles which induce local heat injury to the inner wall of the vein [136]. The heat causes the wall of the vein to shrink, causing the lumen to reduce. This technique is also done with local anesthesia in an outpatient setting. The effectiveness of RFA and EVLA are reported to be about the same. However, RFA had fewer side effects (thrombophlebitis, hyperpigmentation, paresthesia, and bruising) and a more rapid recovery [20].

Foam Sclerotherapy

This is a minimally invasive procedure used to ablate the saphenous vein. The foams are a gas-liquid mixture with surfactant properties used to injure endothelium and trigger coagulation, fibrosis, and vein lumen occlusion [136]. The most common complication with foam sclerotherapy is superficial thrombophlebitis. In a study of 152 geriatric patients with great saphenous vein valvular incompetence and saphenofemoral junction incompetence, the complete occlusion rate of the great saphenous vein 12 months following the procedure was 86.4% with no major complications reported [137]. It seems that surgical treatment, whether invasive or minimally invasive, is a viable option for geriatric patients with severe CVI interested in diminishing their symptoms and improving their quality of life.

## Conclusions

The findings show advanced age to be an important risk factor contributing to CVI and that other age-related factors add to the risk of severe complications. These include pulmonary embolism and venous leg ulcers attributable to compromised venous hemodynamics. The diagnostic differential between CVI and other lower extremity conditions may be more difficult when assessing the elderly because other lower extremity conditions tend to be more prevalent as one age. Furthermore, understanding the mechanism of action of compression therapy, the mainstay of CVI treatment, and its physiological impacts, allows for its informed use in geriatric patients with increased risks of potential compression-related side effects.
